# Inhibition of phosphoinositide 3-kinase/Akt pathway decreases hypoxia inducible factor-1α expression and increases therapeutic efficacy of paclitaxel in human hypoxic gastric cancer cells

**DOI:** 10.3892/ol.2014.1963

**Published:** 2014-03-11

**Authors:** JING ZHANG, HUA GUO, JIN-SHUI ZHU, YU-CHEN YANG, WEI-XIONG CHEN, NI-WEI CHEN

**Affiliations:** Department of Gastroenterology, Shanghai Jiao Tong University Affiliated Sixth People’s Hospital, Shanghai 200233, P.R. China

**Keywords:** phosphoinositide 3-kinase, Akt, hypoxia inducible factor-1α, gastric cancer, paclitaxel, hypoxia

## Abstract

The phosphatidylinositol-3-kinase (PI3K)/Akt signaling pathway plays an important role in cell proliferation, transformation, apoptosis, tumor growth and angiogenesis. Paclitaxel is commonly used to treat multiple human malignancies; however, the underlying mechanisms of paclitaxel in gastric cancer (GC) have not been fully investigated. In the present study, specimens from 45 GC and 36 chronic gastritis patients were collected, and the correlations of PI3K, phosphorylated-Akt (p-Akt) and hypoxia-inducible factor-1α (HIF-1α) expression with the clinicopathological characteristics of GC were analyzed by immunohistochemistry. The human SGC-7901 GC cells under hypoxic conditions were pretreated with the PI3K inhibitor, LY294002 (40 μM), and paclitaxel (0.1 μM). The expression levels of PI3K, p-Akt and HIF-1α were detected by quantitative polymerase chain reaction and western blotting. Cell proliferative activity and apoptosis were evaluated by the Cell Counting Kit-8 assay and flow cytometry. As a result, the rates of positive expression of PI3K, p-Akt and HIF-1α were significantly higher in GC compared with chronic gastritis patients (each P<0.01), and were positively associated with the tumor-node-metastasis (TNM) staging, lymph node metastases, lymphatic infiltration and vascular infiltration (each P<0.01), but inversely correlated with tumor differentiation (P<0.01) in patients with GC. Under hypoxic conditions, the combined inhibition of the PI3K/Akt pathway with paclitaxel markedly reduced the proliferative activity and induced cell apoptosis in GC cells compared with the single treatment of PI3K inhibitor or paclitaxel (each P<0.01), and was accompanied by a decreased expression of HIF-1α. Overall, our findings indicate that the increased expression of the PI3K/Akt/HIF-1α pathway was closely correlated with tumor differentiation, TNM staging, lymph node metastases and lymphatic and vascular infiltration. The inhibition of the PI3K/Akt pathway enhanced the therapeutic efficacy of paclitaxel in GC cells under hypoxic conditions, suggesting that the PI3K/Akt/HIF-1α pathway may act as an important therapeutic target for paclitaxel treatment of GC.

## Introduction

Gastric cancer (GC) is one of the most common types of malignancies worldwide, with an estimated 934,000 cases reported globally in 2002, and is the second most common cause of cancer-related mortality (1). GC is also a genetic disease developing from a multistep process. Single or multiple mutations in genes associated with growth control, invasion and metastasis, form the molecular genetic basis of malignant transformation and tumor progression (2). Chemotherapy, to a certain extent, plays a critical role in the treatment of malignant tumors. However, the identification of predictive biomarkers of resistance or sensitivity to chemotherapy remains a fundamental challenge in the selection of patients most likely to benefit from it.

The phosphatidylinositol-3-kinase (PI3K)/Akt pathway has been shown to be activated in a variety of cancer types. Studies have shown that the increased expression of PI3K or phosphoinositide 3-Akt (p-Akt) contributes to gallbladder carcinogenesis (3) or predicts the survival of advanced endometrial carcinoma (4). Activation of the PI3K/Akt pathway is required for the apoptotic evasion (5) and is significantly associated with increasing tumor grade, decreased apoptosis and clinical outcome in human gliomas (6). It supports the development of metastatic cancer and promotes the aggressive behavior of soft tissue sarcoma (7,8), indicating the PI3K/Akt pathway as an important biomarker for the prognosis of cancer patients.

Hypoxia-inducible factor-1α (HIF-1α) plays an essential role in the adaptive response of cells to hypoxia and is associated with aggressive tumor behavior. HIF-1α is highly expressed in small-cell lung cancer and aids in predicting the overall survival of patients (9), as well as in selecting patients most likely to benefit from HIF-1α-targeted therapies (10). HIF-1α is also overexpressed in mantle cell lymphoma, where it enhances the aggressive potential and, therefore, this observation may result in more efficient target therapies (11). Notably, hypoxia induces a biphasic effect on HIF-1α stabilization with accumulation in early hypoxia, depending on activation of the PI3K/Akt pathway (12). In hypoxic tumor cells, reactive oxygen species increase HIF-1α transcription via the PI3K/Akt pathway (13), and silencing of HIF-1α suppresses tumorigenicity of renal cell carcinoma through the regulation of the PI3K/Akt pathway (14). Activation of PI3K/Akt signaling promotes the progression of hepatocarcinogenesis, while its blockade controls angiogenesis and tumor growth by regulating the expression of HIF-1α (15,16).

Notably, the expression of PI3K/Akt is increased in non-small cell lung cancer treated with adjuvant chemotherapy and serves as a novel independent prognostic biomarker (17). Deregulation of the PI3K/Akt pathway is associated with resistance to the chemotherapeutic agent and confers drug resistance to treatment with paclitaxel in breast cancer (18,19). Although certain studies have demonstrated the enhanced effectiveness of targeting tumor cells with combinations of chemotherapeutic agents and signal transduction inhibitors (20), the enhancing effects of blockade of the PI3K/Akt pathway on paclitaxel in hypoxic GC cells remains unclear. In the present study, the correlations of PI3K, p-Akt and HIF-1α expression with the clinicopathological characteristics of patients with GC were analyzed. Hypoxic GC SGC-7901 cells were pretreated with LY294002 and/or paclitaxel to investigate the enhancing effects of the PI3K inhibitor on paclitaxel through cell proliferation activity and apoptosis.

## Materials and methods

### Materials

The human GC SGC7901 cell line was donated from the Shanghai Tumor Research Institute (no. 01842; Shanghai, China). The primers of PI3K and HIF-1α were synthesized by the Shanghai Biological Engineering Technology Co., Ltd. (Shanghai, China). The PI3K rabbit-anti-human polyclonal antibody (sc-1637) was purchased from Santa Cruz Biotechnology, Inc. (Santa Cruz, CA, USA); p-Akt rabbit-anti-human polyclonal antibody (D9E) and β-actin were purchased from Cell Signaling Technology, Inc. (Danvers, MA, USA); and HIF-1α rabbit-anti-human polyclonal antibody (BA0912) was obtained form Wuhan Boster Biological Engineering Co., Ltd (Wuhan, China). The GC tissue samples treated with paclitaxel were obtained from severe combined immune deficiency (SCID) mice orthotopically implanted with human GC cells from the Gastrointestinal Disease Laboratory of Shanghai Sixth People’s Hospital (Shanghai, China).

### Drugs and reagents

Paclitaxel was donated by Professor G-Y Fan from the Kunming Botany Institute (Yunnan, China); the Cell Counting Kit-8 (CCK-8) was purchased from Tongren Chemical Institute (Japan); Dulbecco’s modified Eagle’s medium (DMEM) and fetal bovine serum (FBS) were purchased from Thermo Fisher Scientific Inc. (Waltham, MA, USA); TRIzol^®^ reagent and LY294002 were obtained from Invitrogen Life Technologies (Carlsbad, CA, USA); M-MLV Reverse Transcriptase was purchased from Promega Corporation (Madison, WI, USA); SYBR Green master mix was purchased from Takara Bio, Inc., (Otsu, Japan); the Annexin V-fluorescein isothiocyanate cell apoptosis detection kit was obtained from KeyGen Biotech., Co., Ltd. (ab14085; Nanjing, China); and the Enhanced Chemiluminescence (ECL)-PLUS™ western blotting reagents were purchased from GE Healthcare (Piscataway, NJ, USA).

### Clinical samples and data

A total of 45 patients with GC and 36 patients with chronic gastritis were enrolled in this study at the General Surgery and Digestive Endoscopy Room from December, 2007 to June, 2008. The pathological staging was determined according to the American Joint Committee on Cancer tumor-node-metastasis (TNM) staging system. The use of the tissue samples and clinical data was approved by the Medical Ethics Committee of Shanghai Jiao Tong University (Shanghai, China).

### Immunohistochemical analysis

The protein expression of PI3K, p-Akt and HIF-1α were analyzed by immunohistochemical staining. The anti-PI3K, p-Akt and HIF-1α antibodies were used at 1:100 dilutions. Endogenous peroxidase was inhibited by incubation with freshly prepared 3% hydrogen peroxide with 0.1% sodium azide. Non-specific staining was blocked with 0.5% casein and 5% normal serum. The tissues were incubated with biotinylated antibodies and horseradish peroxidase (Cell Signaling Technology, Inc.). Staining was developed with diaminobenzidine substrate and sections were counterstained with hematoxylin and eosin. Normal serum or phosphate-buffered saline (PBS) replaced the antibodies in negative controls. The images were analyzed with Image-Pro Plus 4.5 System (Media Cybernetics, Inc., Rockville, MD, USA). The total optical density value and area of intracellular fluorescence for each section was measured.

### Cell culture and pretreatment

The SGC7901 cells were cultured in DMEM supplemented with 10% heat-inactivated FBS, 100 U/ml penicillin and 100 μg/ml streptomycin. The cells were stored in a humidified atmosphere of 5% CO_2_ at 37°C for 30 min. Under normal oxygen, the cells were incubated in 20% O_2_ and 5% CO_2_ with saturated humidity at 37°C for 30 min. The hypoxic cells were incubated in an AnaeroPack™ containing 20% CO_2_ and <1% O_2_ at 37°C for 30 min. The cells pretreated under hypoxic conditions were further treated with the PI3K inhibitor, LY294002, (40 Mm) for 30 min, followed by paclitaxel (0.1 Mm).

### Quantitative polymerase chain reaction (qPCR)

To quantitatively determine the mRNA expression levels of PI3K and HIF-1α in GC SGC-7901 cells, qPCR was used. Total RNA of each clone was extracted with TRIzol reagent according to the manufacturer’s instructions. Reverse-transcription using M-MLV, and cDNA amplification using SYBR Green master mix kit, were performed according to the manufacturer’s protocol. The genes were amplified using specific oligonucleotide primers and a human β-actin gene was used as an endogenous control. The PCR primer sequences were as follows: Forward, 5′-AAGAAGCAAGCAGCTGAG-3′ and reverse, 5′-CTACAGAGCAGGCATAG-3′ for PI3K; forward, 5′-TCAAAGTCGGACAGCCTC-3′ and reverse, 5′-CCAGCAGTCTACATGC-3′ for HIF-1α; forward, 5′-CTTCGAGCAAGAGATGGC-3′ and reverse, 5′-CTCCTTCTGCATCCTGTC-3′ for β-actin. Data were analyzed using the comparative Ct method (2^−ΔΔCt^). Three separate experiments were performed for each clone.

### Western blot analysis

The hypoxic SGC-7901 cells treated with LY294002 and/or paclitaxel were harvested and extracted using lysis buffer [Tris-HCl, sodium-dodecyl sulfate (SDS), mercaptoethanol and glycerol] purchased from Santa Cruz Biotechnology, Inc. Cell extracts were heated to boiling point for 5 min in loading buffer and equal amounts of cell extracts were separated on 15% SDS-PAGE gels. Separated protein bands were transferred into polyvinylidene fluoride membranes, which were blocked in 5% skimmed milk powder. The primary antibodies against PI3K, p-Akt and HIF-1α were diluted according to the manufacturer’s instructions and incubated overnight at 4°C. Horseradish peroxidase-linked secondary biotinylated antibodies were added at a dilution ratio of 1:1,000 and incubated at room temperature for 2 h. The membranes were washed with PBS three times and the immunoreactive bands were visualized using the ECL-PLUS/Kit according to the manufacturer’s instructions. The relative protein levels in different cell lines were normalized to the GAPDH concentration. Three separate experiments were performed for each clone. Finally, the immune complexes were developed using an ECL detection kit according to the manufacturer’s instructions (ECL GST western blotting detection kit, Pierce Biotechnology, Inc., Waltham, MA, USA) and the GelGDoc2000 imaging system (Bio-Rad Laboratories GmbH, Munich, Germany) was employed to analyze the bands, and the protein levels by the relative optical density.

### CCK-8 assay

Cell proliferation following treatment with LY294002 and/or paclitaxel was measured by the CCK-8 assay. The GC SGC-7901 cells were seeded at a density of 2×10^4^ cells/100 μl/well in 96-well plates and left to attach overnight. The medium was then removed and 200 μl of FBS was added followed by LY294002 and/or paclitaxel. The cells were incubated under these conditions for 24, 48, 72, 96, 120 and 148 h at 37°C in a humidified atmosphere of 5% CO_2_. After the designated time, CCK-8 was added to each well containing 200 μl of the culture medium and the oligopeptide mixture, and further incubated for 4 h at 37°C. The amount of formazan dye was measured at 450 nm using the Multi-Mode Microplate Reader (Nanchang Biotek Medical Device Co., Ltd., Nanchang, China). All the experiments were performed in triplicate and repeated three times.

### Cell apoptosis analysis

Cell apoptosis detection was performed using the BD Accuri™ C6 Flow cytometer (BD Biosciences, San Jose, CA, USA). The exposure of PBS on the extracellular side of the cell membrane was quantified by propidium iodide (PI) staining (Invitrogen Life Technologies). The SGC-7901 cells were placed in six-well plates and, after 24 h of incubation, the cells were treated with LY294002 and/or paclitaxel for 24 h and then harvested. Following centrifugation, cell pellets were washed twice with cold PBS. The cells were then incubated with 5 μl of PI at room temperature for 15 min in the dark. Following incubation, 400 μl of 1X binding buffer was added to each tube. The cells were immediately analyzed by flow cytometry.

### Statistical analysis

Data are expressed as the means ± standard deviation where applicable. Statistically significant differences in each assay were determined by SPSS software, version 20.0 (SPSS, Inc., Chicago, IL, USA). Differences in each group were tested for significance using Student’s t-test or one-way analysis of variance. P<0.05 was considered to indicate a statistically significant difference.

## Results

### Correlations of PI3K, p-Akt and HIF-1α expression with the clinicopathological characteristics

**Figure 1:**
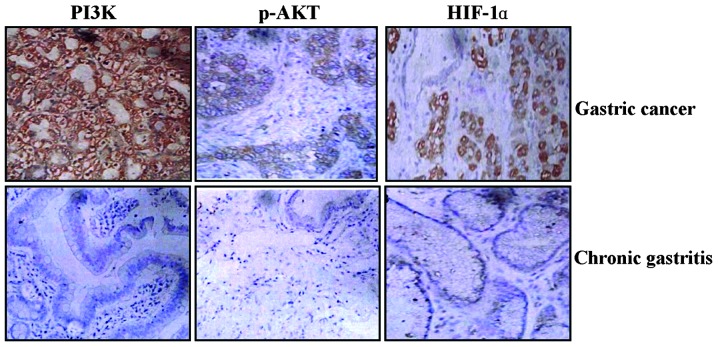
Expression of PI3K, p-AKT and HIF-1α in GC tissues (magnification, ×200). The positive expression of PI3K, p-AKT and HIF-1α was predominantly localized in the cytoplasm of GC tissue cells but was rarely identified in chronic gastritis, and their expression intensities were significantly increased in GC tissue compared with chronic gastritis tissue. PI3K, phosphatidylinositol-3-kinase; p-AKT, phosphorylated-Akt; HIF-1α, hypoxia-inducible factor-1α; GC, gastric cancer.

The GC tissue sections were analyzed by immunohistochemistry (IHC) and Image-Pro Plus 4.5 software (Media Cybernetics, Inc.). As shown in Fig. 1 and Table I, the positive expression of PI3K, p-Akt and HIF-1α was predominantly localized in the cytoplasm of the GC tissue cells, but was not identified in the chronic gastritis cells. The expression intensities of PI3K, p-Akt and HIF-1α were significantly increased in GC tissues compared with chronic gastritis tissues (each P<0.01).

The correlations of PI3K, p-Akt and HIF-1α expression and various clinical and pathological characteristics were analyzed. As summarized in Table II, no significant correlation was found between PI3K, p-Akt and HIF-1α expression and gender, age, tumor size and peripheral nerve infiltration in patients with GC (each P>0.05), while their expression was significantly correlated with TNM staging, lymph node metastases, lymphatic infiltration and vascular infiltration (each P<0.01), but inversely correlated with tumor differentiation (P<0.01).

### Effects of LY294002 and/or paclitaxel on the expression of PI3K, p-Akt and HIF-1α

**Figure 2:**
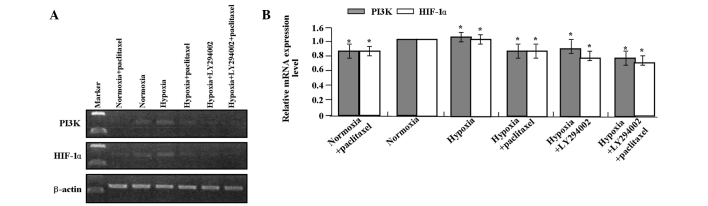
Effects of LY294002 and/or paclitaxel on the mRNA expression of PI3K and HIF-1α. (A) Quantitative polymerase chain reaction was performed to detect the mRNA expression levels. (B) LY294002 combined with paclitaxel markedly reduced the mRNA expression levels of PI3K and HIF-1α compared with the single treatment of LY294002 or paclitaxel (each P<0.01). LY294002 or paclitaxel decreased the RNA expression levels of PI3K and HIF-1α compared with the hypoxia group. ^*^P<0.01 compared with normoxia. PI3K, phosphatidylinositol-3-kinase; HIF-1α, hypoxia-inducible factor-1α.

**Figure 3:**
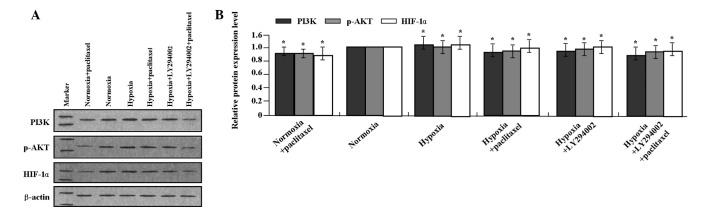
Effects of LY294002 and/or paclitaxel on the protein expression of PI3K, p-AKT and HIF-1α. (A) Western blotting was performed to detect the protein expression levels. (B) LY294002 combined with paclitaxel significantly decreased the protein expression levels of PI3K, p-AKT and HIF-1α compared with the single treatment of LY294002 or paclitaxel (each P<0.01). LY294002 or paclitaxel decreased the protein expression of PI3K, p-AKT and HIF-1α compared with the hypoxia group. ^*^P<0.01 compared with normoxia. PI3K, phosphatidylinositol-3-kinase; p-AKT, phosphorylated-Akt; HIF-1α, hypoxia-inducible factor-1α.

qPCR and western blot analysis were performed to detect the effects of LY294002 and paclitaxel on the expression of PI3K, p-Akt and HIF-1α in the GC SGC-7901 cells. LY294002 combined with paclitaxel markedly inhibited the mRNA (Fig. 2A and B) expression levels of PI3K and HIF-1α (it was not necessary to detect the expression of p-Akt as it is downstream of PI3K) and protein (Fig. 3A and B) expression levels of PI3K, p-Akt and HIF-1α in GC SGC-7901 cells compared with the single treatment of LY294002 or paclitaxel (each P<0.01). LY294002 or paclitaxel decreased the expression of PI3K, p-Akt and HIF-1α at the transcriptional and translational levels compared with the hypoxic group (each P<0.01).

### Effects of LY294002 and/or paclitaxel on cell proliferation

To determine whether LY294002 and/or paclitaxel affect the proliferative activity of hypoxic GC cells, the CCK-8 assay was performed to detect cell viability. As summarized in Table III, LY294002 combined with paclitaxel significantly decreased the cell viability in SGC-7901 cells compared with the single treatment of LY294002 or paclitaxel (each P<0.01). In addition, LY294002 or paclitaxel reduced cell viability compared with the hypoxic group (each P<0.01).

### Effects of LY294002 and/or paclitaxel on cell apoptosis

**Figure 4:**
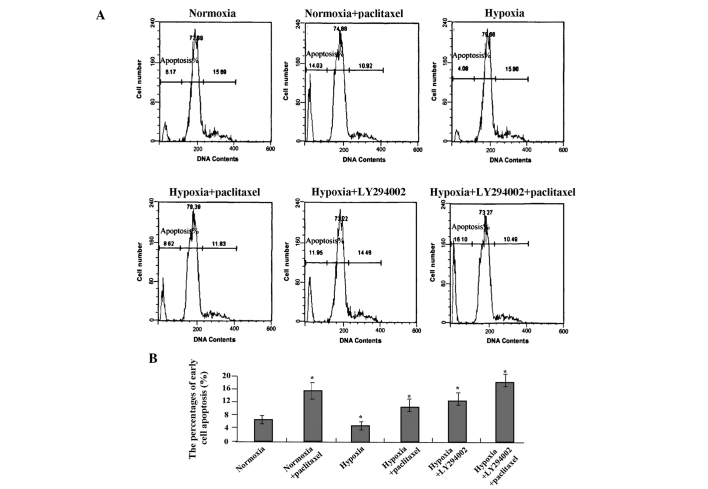
Effects of LY294002 and/or paclitaxel on cell apoptosis. (A) Cell apoptosis was measured by flow cytometry analysis using propidium iodide staining. (B) Following treatment with LY294002 and/or paclitaxel for 24 h, LY294002 combined with paclitaxel significantly increased the percentage of early cell apoptosis compared with the single treatment of LY294002 or paclitaxel (each P<0.01). LY294002 or paclitaxel also improved the percentage of early cell apoptosis compared with the hypoxia group. ^*^P<0.01 compared with normoxia.

Cell apoptosis was measured by flow cytometry using PI staining. Following treatment with LY294002 and/or paclitaxel for 24 h, LY294002 combined with paclitaxel significantly increased the percentage of early cell apoptosis compared with the single treatment of LY294002 or paclitaxel (each P<0.01) (Fig. 4A and B). LY294002 or paclitaxel increased the percentage of early cell apoptosis compared with the hypoxic group (both P<0.01).

### Effects of paclitaxel on the expression of PI3K, p-Akt and HIF-1α in vivo

**Figure 5:**
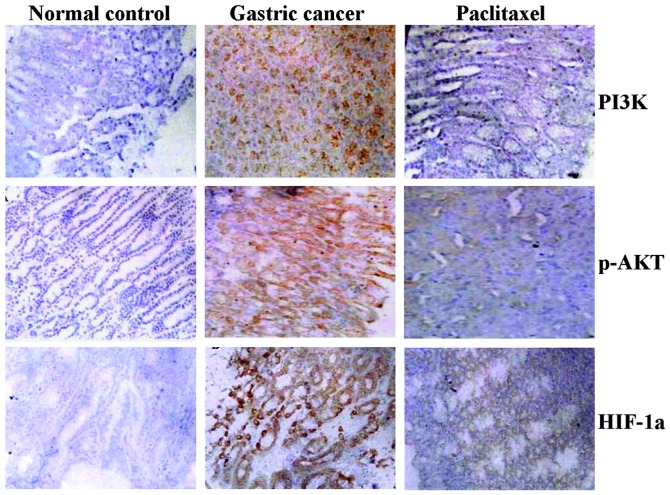
Effects of paclitaxel on the expression of PI3K, p-AKT and HIF-1α *in vivo*. The expression of PI3K, p-AKT and HIF-1α were assessed by immunohistochemistry in severe combined immune deficiency mice orthotopically implanted with human GC tissues and treated with paclitaxel. Paclitaxel decreased the expression of PI3K, p-AKT and HIF-1α compared with the GC and normal control groups. PI3K, phosphatidylinositol-3-kinase; p-AKT, phosphorylated-Akt; HIF-1α, hypoxia-inducible factor-1α.

SCID mice orthotopically implanted with human GC tissues were treated with paclitaxel (intraperitoneal administration of 5 mg/kg) and the effects of paclitaxel on the expression of PI3K, p-Akt and HIF-1α were assessed by IHC. Paclitaxel decreased the expression of PI3K, p-Akt and HIF-1α (Fig. 5) compared with the GC and normal control groups (each P<0.01).

## Discussion

The PI3K/Akt pathway is one of the most important signaling networks in cancer. Emerging evidence indicates that activation of this pathway plays a significant role in the development and progression of certain malignancies. Zhang *et al* reported that the PI3K/Akt pathway is expressed in 71.7 (43/60) and 68.3% (41/60) of colon cancer and is closely associated with serous coat infiltration and lymphatic metastasis (21), serving as an independent prognostic marker for patients with colorectal cancer (CRC) (22). HIF-1α is a transcription factor recognized to control the delivery of oxygen and nutrients through the induction of angiogenesis under hypoxic conditions. Overexpression of HIF-1α is significantly correlated with histology, depth of invasion and poor prognosis for patients with GC, and may be utilized for tumor-specific molecular target-based therapy (23). In the present study, the positive expression of PI3K, p-Akt and HIF-1α were significantly increased in GC tissues compared with chronic gastritis, and were associated with TNM staging, lymph node metastases and lymphatic and vascular infiltration, but inversely correlated with tumor differentiation. This suggested that PI3K, p-Akt and HIF-1α are potential therapeutic targets for GC.

Inhibiting the molecules involved in the PI3K/Akt or HIF-1α signal transduction pathway is a possible strategy for the treatment of cancer. The PI3K inhibitor and conventional chemotherapy provide an effective approach to inhibiting tumor growth in ovarian cancer (24). Paclitaxel is extensively used in chemotherapy for various cancers. PI3K is involved in low susceptibility of CRC to paclitaxel and PI3K-targeting agents may enable a new paclitaxel-based chemotherapy for CRC (25). Furthermore, the Akt inhibitor increases the therapeutic efficacy of paclitaxel for patients with ovarian cancer (26). HIF-1α affects the sensitivity of paclitaxel in lung cancer cells and targeted inhibition of HIF-1α may overcome the drug resistance of paclitaxel (27). However, an individual study has reported that pharmacological PI3K/Akt inhibition antagonizes the efficacy of chemotherapeutic agents primarily effective in the S or G2 phase of the cell cycle (28). Our current study indicated that combining the PI3K inhibitor, LY294002, with paclitaxel significantly decreased cell viability and increased cell apoptosis in hypoxic GC SGC-7901 cells compared with the single treatment of LY294002 or paclitaxel. Moreover, treatment with LY294002 or paclitaxel reduced cell viability and induced apoptosis compared with the hypoxic GC cells, suggesting that PI3K/Akt-targeted therapy with paclitaxel is more efficacious for treating GC with activated PI3K/Akt signaling.

Notably, the PI3K/Akt pathway regulates HIFα activity (29), and HIF-1α-dependent transcription is blocked by negative Akt or PI3K and by the wild-type phosphatase and tensin homolog (30). Direct evidence has also revealed that activation of the PI3K/Akt/HIF-1α pathway plays a critical role in mediating hypoxia-induced drug resistance resulting in an unfavorable treatment outcome of hepatocellular carcinoma (31). However, Manohar *et al* (32) reported that the HIF-1α inhibitor modulates the PI3K/Akt pathway and contributes to antitumor activity. In the present study, LY294002 combined with paclitaxel markedly inhibited the expression of PI3K, p-Akt and HIF-1α in GC SGC-7901 cells compared with the single treatment of LY294002 or paclitaxel, and LY294002 or paclitaxel decreased the expression of PI3K and p-Akt in hypoxic conditions, suggesting that targeting PI3K/Akt signaling in tumor cells may inhibit HIF-1α expression and increase the therapeutic efficacy of paclitaxel.

In conclusion, our findings suggest that the increased expression of the PI3K/Akt or HIF-1α pathway is closely correlated with tumor differentiation, TNM staging, lymph node metastases, and lymphatic and vascular infiltration. In addition, inhibition of the PI3K/Akt pathway enhanced the therapeutic efficacy of paclitaxel in GC cells under hypoxic conditions, suggesting that the PI3K/Akt or HIF-1α pathway may serve as an important therapeutic target for the paclitaxel treatment of cancer.

## Figures and Tables

**Table I:** Expression of PI3K, p-AKT and HIF-1α in gastric cancer (Gray values).

Markers	Group	Cases	Gray value	P-value
PI3K	Gastric cancer	45	202.4±10.7	<0.01
	Chronic gastritis	36	220.3±4.3	
p-AKT	Gastric cancer	45	223.6±11.7	<0.01
	Chronic gastritis	36	234.0±4.6	
HIF-1α	Gastric cancer	45	200.3±6.9	<0.01
	Chronic gastritis	36	218.7±4.4	

PI3K, phosphatidylinositol-3-kinase; p-AKT, phosphorylated-Akt; HIF-1α, hypoxia-inducible factor-1α.

**Table II:** Correlation of PI3K, p-AKT and HIF-1α expression with the clinicopathological characteristics of patients with GC.

Variables	Cases	PI3K	P-value	p-AKT	P-value	HIF-1α	P-value
Age, years							
≤68	23	201.0±9.2	0.052	223.7±10.7	0.841	200.7±6.8	0.335
>68	22	203.9±10.3		223.4±11.7		199.8±7.0	
Gender							
Male	32	202.9±10.9	0.279	223.9±11.0	0.526	200.7±6.6	0.142
Female	13	201.2±10.4		223.0±11.7		199.2±7.4	
Tumor size, cm							
≤5	30	202.4±8.4	0.926	224.2±10.8	0.256	200.8±5.4	0.184
>5	15	202.9±8.4		200.3±6.9		199.3±9.1	
Degree of differentiation							
Well/moderately	15	206.3±10.9	<0.01	227.6±10.6	<0.01	202.1±6.3	0.005
Poorly	30	200.4±10.2		221.6±10.9		199.4±7.0	
TNM staging							
I+II	20	207.7±8.8	<0.01	228.6±9.5	<0.01	204.3±5.6	<0.01
III+IV	25	198.1±10.3		219.6±10.8		197.1±6.1	
Lymph node metastases							
No	19	208.7±8.4	<0.01	228.7±9.6	<0.01	204.3±5.7	<0.01
Yes	26	197.8±10.2		219.8±10.7		197.3±6.1	
Lymphatic vessel infiltration							
−	11	207.6±7.0	<0.01	230.7±8.4	<0.01	205.7±5.3	<0.01
+	34	200.7±11.2		221.3±11.0		198.5±6.4	
Vascular infiltration							
−	29	205.0±9.9	<0.01	226.6±10.6	<0.01	201.6±7.2	<0.01
+	16	197.6±10.4		218.1±10.0		197.9±5.5	
Perineural infiltration							
−	6	203.6±6.6	0.324	225.7±10.4	0.265	202.5±4.8	0.053
+	39	202.2±11.3		223.2±11.3		199.9±7.1	

PI3K, phosphatidylinositol-3-kinase; p-AKT, phosphorylated-Akt; HIF-1α, hypoxia-inducible factor-1α; TNM, tumor-node-metastasis.

**Table III:** Proliferative activity of gastric cancer cells (optical density values).

	Day					
	
Groups	1	2	3	4	5	6
Normoxia	0.45±0.01	0.75±0.02	1.25±0.06	1.60±0.04	1.86±0.04	2.12±0.10
Normoxia+paclitaxel	0.43±0.02	0.60±0.03^a^	0.87±0.0^a^	1.22±0.04^a^	1.54±0.05^a^	1.74±0.07^a^
Hypoxia	0.46±0.03	0.68±0.04^a^	1.05±0.05^a^	1.40±0.07^a^	1.65±0.02^a^	1.85±0.04^a^
Hypoxia+paclitaxel	0.44±0.06	0.59±0.04^a^,^b^	0.83±0.05^a^,^b^	1.11±0.05^a^,^b^	1.38±0.07^a^,^b^	1.60±0.10^a^,^b^
Hypoxia+LY294002	0.45±0.04	0.64±0.03^a^–^c^	0.85±0.02^a^–^c^	1.10±0.10^a^,^b^	1.33±0.12^a^–^c^	1.61±0.11^a^–^c^
Hypoxia+paclitaxel+LY294002	0.42±0.07	0.53±0.03^a^–^c^	0.67±0.03^a^–^c^	0.87±0.03^a^–^c^	1.11±0.12^a^–^c^	1.29±0.04^a^–^c^

a P<0.01, vs. normoxia;

b P<0.01, vs. hypoxia;

c P<0.01, vs. hypoxia+paclitaxel.
